# Exploring the Associations Between Self-reported Tendencies Toward Smartphone Use Disorder and Objective Recordings of Smartphone, Instant Messaging, and Social Networking App Usage: Correlational Study

**DOI:** 10.2196/27093

**Published:** 2021-09-30

**Authors:** Davide Marengo, Rayna Sariyska, Helena Sophia Schmitt, Eva-Maria Messner, Harald Baumeister, Matthias Brand, Christopher Kannen, Christian Montag

**Affiliations:** 1 Department of Psychology University of Turin Turin Italy; 2 Department of Molecular Psychology Institute of Psychology and Education Ulm University Ulm Germany; 3 Department of Clinical Psychology and Psychotherapy Institute of Psychology and Education Ulm University Ulm Germany; 4 Department of General Psychology: Cognition and Center for Behavioral Addiction Research (CeBAR) Faculty of Engineering University of Duisburg-Essen Duisburg Germany

**Keywords:** smartphone use disorder, smartphone use, social media, objective measures, mediation model, smartphone, web-based communication, social networking, mobile phone

## Abstract

**Background:**

Social communication via instant messaging (IM) and social networking (SN) apps makes up a large part of the time that smartphone users spend on their devices. Previous research has indicated that the excessive use of these apps is positively associated with problematic smartphone use behaviors. In particular, image-based SN apps, such as Instagram (Facebook Inc) and Snapchat (Snap Inc), have been shown to exert stronger detrimental effects than those exerted by traditional apps, such as Facebook (Facebook Inc) and Twitter (Twitter Inc).

**Objective:**

In this study, we investigated the correlation between individuals’ tendencies toward smartphone use disorder (SmUD) and objective measures of the frequency of smartphone usage. Additionally, we put to test the hypothesis that the pathway linking the frequency of actual smartphone usage to self-reported tendencies toward SmUD was mediated by the increased frequency of IM and SN app usage.

**Methods:**

We recruited a sample of 124 adult smartphone users (females: 78/124, 62.9%; age: mean 23.84 years, SD 8.29 years) and collected objective information about the frequency of smartphone and SN app usage over 1 week. Participants also filled in a self-report measure for assessing the multiple components of tendencies toward SmUD. Bivariate associations were investigated by using Spearman correlation analyses. A parallel mediation analysis was conducted via multiple regression analysis.

**Results:**

The frequency of smartphone usage, as well as the use of IM apps (Messenger, Telegram, and WhatsApp [Facebook Inc]), Facebook, and image-based apps (Instagram and Snapchat), had significant positive associations with at least 1 component of SmUD, and the cyberspace-oriented relationships factor exhibited the strongest associations overall. We found support for an indirect effect that linked actual smartphone usage to SmUD tendencies via the frequency of the use of image-based SN apps.

**Conclusions:**

Our novel results shed light on the factors that promote SmUD tendencies and essentially indicate that image-based SN apps seem to be more strongly associated with problematic smartphone behaviors compared to IM apps and traditional SN apps, such as Facebook.

## Introduction

Web-based social communication via instant messaging (IM) and social networking (SN) apps makes up a large part of the time that smartphone users spend on their devices, and this time exceeds the time that smartphone users spend on other activities, including being productive and gaming [1]. Among the most popular IM apps are WhatsApp (Facebook Inc), Facebook Messenger (Facebook Inc), WeChat (Tencent Holdings Limited), Telegram, and Signal (Signal Messenger LLC), with WhatsApp being the most popular IM app worldwide [2]. IM apps allow for the one-to-one and group-based transmission of messages and vary considerably in terms of their specific characteristics and affordances (eg, file sharing and voice calling). Popular SN apps include Facebook (Facebook Inc), Instagram (Facebook Inc), Twitter (Twitter Inc), Snapchat (Snap Inc), and TikTok, with Facebook being the most popular SN app [3]. SN apps feature microblogging capabilities that allow smartphone users to broadcast messages, pictures, or videos to an extensive network of smartphone users and receive feedback (ie, comments and likes) on the shared content—a feature that is distinctive from those offered by IM apps and may play a crucial role in triggering problematic behaviors [4,5]. Currently, SN apps are shifting toward image-based ephemeral media sharing (ie, media that are available for a limited time)—a feature that was introduced by image-based apps such as Snapchat and Instagram but has also been implemented on more traditional platforms (eg, the recent introduction of Facebook’s time-limited stories feature). Notably, such a time restriction on content availability might pressure smartphone users to watch or read a story instantly. Otherwise, one might miss crucial information in one’s social network.

Previous findings have indicated that the excessive use of SN apps on smartphones is positively associated with problematic behaviors related to the use of devices in general [6-8]. These findings suggest that the link between problematic smartphone use and smartphone use disorder (SmUD; a discussion of terminology was conducted by Montag and colleagues [9] in 2021) can, in part, be explained by the use and overuse of SN apps on smartphones. It is worthy to note that many of the available inventories that assess problematic behaviors in this area of research use an addiction framework. However, the question regarding the actual nature of problematic smartphone and SN use has not been ultimately settled until now. In line with the term *disorder*, which is used to describe behavioral addictions in the *International Classification of Diseases, 11th edition* (eg, gaming disorder) as well as those in previous literature related to this area of research [7,10], we chose to use the term SmUD in this paper. Still, we explicitly stress the importance of refraining from pathologizing everyday life behaviors [11]. Moreover, we explicitly mention that so far, SmUD does not represent an official diagnosis, and in this paper, we aimed to unify the terminology used in related literature without judging on the nature of the construct underlying the term itself. It must also be mentioned that the concept of SmUD is being fiercely discussed by researchers, as smartphones are only vehicles for accessing various apps, which in turn may trigger the addiction to the device itself [12], similar to how someone diagnosed with an alcohol use disorder is not dependent on the bottle but on the beverage. Beyond this, it is very likely that for many smartphone users, SmUD might resemble social networks use disorder to a high degree due to the substantial overlap of concepts [7,13]. Further, we also want to hint at recent works that discuss what prerequisites need to be met to speak of a disorder that results from problematic behaviors and might be classified within the residual category of “other specified disorders due to addictive behaviors” in the *International Classification of Diseases, 11th edition*. The criteria suggested are (1) scientific relevance for clinical relevance, (2) theoretical embedding within addiction frameworks, and (3) empirical evidence for the validity of mechanisms that underlie addictive behaviors. In terms of a potential social networks use disorder, these criteria are widely met, although the scientific evidence for clinical relevance and functional impairment are still less convincing compared to the evidence for other disorders resulting from addictive behaviors, such as gaming disorder [14].

It is noteworthy that although we distinguish between messaging and SN apps in this paper, SN can be seen as an umbrella term that also includes messenger apps [15]. For our research, it is of interest that increasing amounts of evidence indicate that image-based apps such as Instagram and Snapchat may exert stronger detrimental effects on adverse health-related outcomes, including sleep problems, psychological distress, and problematic behaviors, compared to those exerted by more traditional SN platforms such as Facebook and Twitter [16-18]. Interestingly, a new study by Rozgonjuk et al [19] reported that the problematic use of WhatsApp and Instagram was the factor that was the most strongly linked to productivity loss in everyday life (ie, compared to Facebook and Snapchat use). Another work has also shown that these two platforms might be associated with the most problematic consequences for smartphone users [20]. These studies are based on self-report data; however, recent literature has explored the associations between smartphone and IM and SN app usage and SmUD based on objective measures [17,21,22] (a related review was conducted by Ryding and Kuss [23]). Some of the studies in this area of research focus on overall measures of smartphone usage, such as screen time and screen unlock events, but do not explore app usage measures [24,25]. Additionally, other studies have provided evidence of the links between SmUD variables and broad app usage categories (eg, chatting [26] and SN use [21,22]). We could only find 1 study [17] that distinguished between traditional and image-based SN platforms when examining the link between app usage and SmUD. The results provided by Noë and colleagues [17] showed that Snapchat use was more strongly related to self-reported tendencies toward SmUD than the use of other IM and SN apps (eg, WhatsApp and Facebook)—a finding that the authors interpreted as the result of the in-built design features in Snapchat that promote high-frequency usage. The findings of Rozgonjuk et al [20] differ from those of Noë et al [17]; by using self-report data, Rozgonjuk et al [20] reported that the lowest magnitude of correlations was found between Snapchat use and SmUD incidence and that the highest correlations were found between SmUD and problematic WhatsApp and Instagram use. Nevertheless, both of these findings point toward the need to distinguish between IM, traditional, and image-based SN apps when examining their associations with SmUD.

To build on these findings, in this study, we aimed to examine the existing associations between objective records of smartphone, IM platform, and SN platform activity and individual differences in self-reported SmUD. More specifically, we tested the following hypotheses:

- Hypothesis 1: Significant associations exist between self-reported tendencies toward SmUD and objective measures of the usage of the smartphone and IM and SN platforms.
- Hypothesis 2: The association between self-reported tendencies toward SmUD and overall smartphone usage is mediated by the frequency of IM and SN app usage (both traditional and image-based apps).

Based on the existing literature about theoretical considerations and previous empirical findings [6-8], we expected that a positive link would emerge between actual smartphone usage and users’ perceptions of being at risk for SmUD. We also expected that this link might, in part, be mediated by the usage of both IM and SN apps and that image-based SN apps would have a stronger mediation effect [17].

## Methods

### Sample

Our sample consisted of individuals who were recruited among students at a German university campus by using a convenience sampling strategy. The inclusion criteria for this study were legal age (18 years and over) and current ownership of a smartphone. Participants were recruited between March 2017 and August 2018 and consisted of 124 individuals with a mean age of 23.84 years (SD 8.29 years; range 18-63 years) and a majority of female individuals (78/124, 62.9%). Participants gained university credits or monetary compensation for their participation. With regard to education level, of the 124 participants, 2 (1.6%) reported having finished secondary school, 10 (8.1%) reported holding a vocational baccalaureate diploma, 92 (74.2%) reported holding an A-level diploma, 3 (2.4%) reported holding a degree from a university of applied sciences, and 17 (13.7%) reported holding a university degree. Prior to their participation in this study, participants were provided with detailed information on the aim of this study, all variables that were tracked by the Insights app (see also the *Objective Smartphone Activity Data* section), how data were recorded, who was granted access to their data, and where data were saved and analyzed. Participants were informed about their right to request the deletion of their data at any time during this study. All participants provided digital informed consent via the app prior to their participation in this study. In order to facilitate the installation process, participants were invited to the laboratory, where scientists guided the participants through the Insights app installation process. Therefore, no problems with the installation process were observed. The study was approved by the local ethics committee of Ulm University, Ulm, Germany. It should be noted that in this study’s data set, there was an overlap among other publications—there was 1 study that investigated links between call variables and personality [27]; 1 study that investigated the molecular genetics of social network size [28]; and 1 study that investigated links between smartphone variables and self-reported mood, drive, and stress levels [29].

### Objective Smartphone Activity Data

In order to collect participants’ smartphone activity data, the Insights app [30]—an Android-based smartphone app—was installed on participants’ phones by either the examiner or the participants themselves. The app allowed for the recording of different variables, such as the number of calls per day (incoming, outgoing, and missed calls), the frequency of specific smartphone events (eg, screen unlock and screen on events), and data related to users’ sessions on smartphone apps. An in-depth review of the Insights app was conducted by Montag et al [27].

Participants varied considerably in terms of the duration of study participation, which ranged from 1 week to 2 months (based on recorded activity). Based on considerations about the potential incomparability of data that are collected during different time frames and the need to maximize our sample size for this study, we decided to limit our analyses to smartphone data that were collected during the first week (7 days) of smartphone data collection. Given this time frame, we retrieved the following information: the number of smartphone sessions that were started by the user unlocking the smartphone screen (ie, the number of screen unlock events) and the number of sessions on IM apps (ie, Facebook Messenger, Telegram, and WhatsApp), traditional SN apps (ie, Facebook and Twitter), and image-based SN apps (ie, Instagram, Snapchat, and TikTok). Participants were coded as having 0 sessions on the app if they either had the app installed on their smartphones and did not use it or did not have the app installed on their smartphones. We followed such a strategy to conduct analyses on the complete sample and to take into account that not each app of interest was installed on each phone. The Insights app allowed for the tracking of both the screen unlock events and screen on events of Android smartphones; each event represented an indicator of users’ active use of their smartphone. However, while both screen unlock events and screen on events were triggered through users’ interaction with their smartphone’s hardware (ie, the screen or buttons), screen on events could also be triggered by app notifications (depending on the smartphone settings). Due to this potential confounding factor, for the purpose of this paper, analyses were presented by using the screen unlock event variable. However, for full transparency, we presented and commented on the associations between screen on and screen unlock events and app session and SmUD variables in Multimedia Appendix 1.

Based on the collected data, WhatsApp was the most used app during the investigated time frame (participants: 122/124, 98.4%), followed by Facebook (70/124, 56.5%), Messenger (60/124, 48.8%), Instagram (56/124, 45.2%), Snapchat (41/124, 33.1%), and Telegram (13/124, 10.50%). Of the 124 participants, 123 (99.2%) used IM apps and 67 (54%) used image-based SN apps. In turn, we found that no participants used the TikTok app; however, it should be noted that at the time data collection started for the present study, the TikTok app was called “musical.ly” [31], and was later renamed TikTok starting on November 2017. Further, only 3 participants reported using the Twitter app. Due to the small number of users, the TikTok and Twitter apps were not considered further in this study.

### Individual Differences in Tendencies Toward SmUD

We administered a German version of the Smartphone Addiction Scale (SAS) [32], which consists of 33 items that are rated on a 6-point Likert scale (1=strongly disagree; 6=strongly agree). It is worthy to note that the SAS scale is not a diagnostic tool for SmUD but is instead meant to provide an assessment of users’ addictive tendencies toward using smartphones. To keep in line with previous studies [7,10] however, in this study, the SAS total score was treated as an indicator of a general tendency toward SmUD. The SAS items assess 6 components of smartphone addiction, namely daily life disturbances (eg, “Missing planned works due to smartphone usage”), positive anticipation (eg, “Feeling pleasant or excited while using a smartphone”), withdrawal (eg, “Having my smartphone in my mind even when I’m not using it”), cyberspace-oriented relationships (eg, “Feeling that my smartphone buddies understand me better than my real-life friends”), overuse (eg, “Using my smartphone longer than I had intended”), and tolerance (eg, “The people around me tell me that I use my smartphone too much”). The items were summed to compute an overall indicator of the tendency toward SmUD (SAS total score: mean 70.10, SD 22.84). For the purpose of this study, the SAS total score had excellent reliability (Cronbach α=.94). The reliability of the 6 components was also adequate (daily life disturbances: α=.80; positive anticipation: α=.80; withdrawal: α=.80; cyberspace-oriented relationships: α=.77; overuse: α=.69; tolerance: α=.86). It should be noted that for most of the participants (74/124, 59.7%), the SAS was administered within 1 month of the beginning of smartphone data collection; the remaining participants filled in the SAS within 6 months (14/124, 11.3%) or more than 6 months (36/124, 29%) from the beginning of smartphone data collection. In regression analyses, we controlled for this effect by using a covariate that represented the time (in months) between self-reports and smartphone data collection.

### Strategy of Analysis

First, we computed descriptive statistics (the mean and SD of the number of app and smartphone sessions). Second, we calculated Spearman correlation values between the number of app and smartphone sessions and age and between the number of such sessions and indicators of tendencies toward SmUD (ie, SAS subscales and total score). Mann-Whitney U tests were performed to evaluate the association between gender and smartphone and app session variables. In all inferential tests, results were considered significant when *P* was <.05.

**Figure 1:**
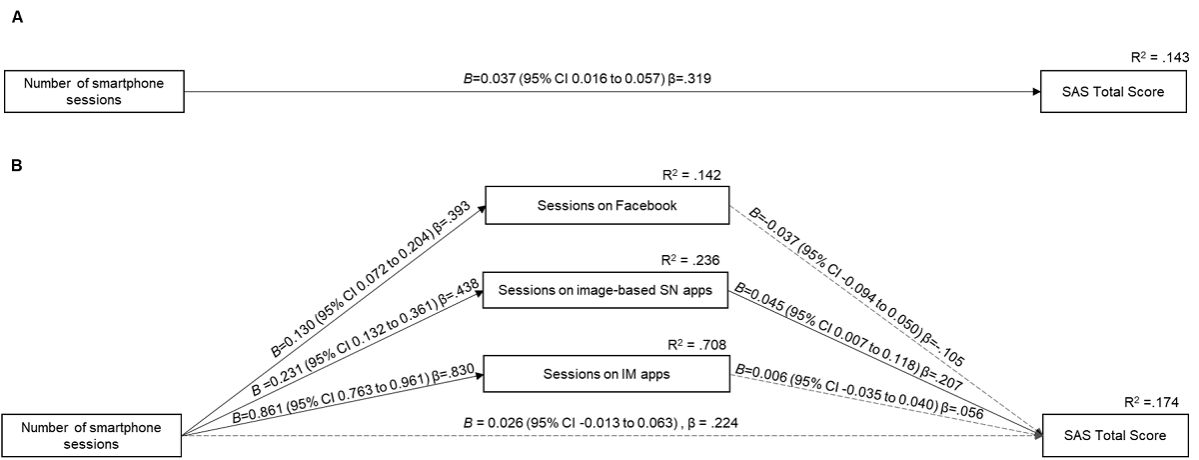
Total effect (A) and mediation (B) models for smartphone use disorder. For ease of visualization, the results of covariates are not reported in the figure. IM: instant messaging; SAS: Smartphone Addiction Scale; SN: social networking.

Third, we performed a mediation analysis via multiple linear regression models for predicting the overall tendency toward SmUD. The diagrams for the examined models are shown in Figure 1. As a first step, we examined the total effect that participants’ frequency of smartphone use (ie, the number of smartphone sessions started by a screen unlock) had on tendencies toward SmUD. In the mediation model, participants’ number of sessions on Facebook, image-based SN apps (ie, Instagram and Snapchat), and IM apps (ie, WhatsApp, Telegram, and Messenger) were investigated as multiple parallel mediators of the link between the frequency of smartphone use (ie, the number of smartphone sessions started by a screen unlock) and tendencies toward SmUD. In all analyses, we controlled for the effects of gender, age, and the time of the administration of the SAS (ie, the number of months after the collection of smartphone data). Analyses were performed by using 10,000 bootstrap samples to compute 95% CIs. Bootstrapping was used, as it did not impose assumptions on the distribution of residuals. Moreover, the use of bootstrapping is appropriate when potentially dealing with nonnormally distributed variables [33]. Estimated effects were deemed significant if 95% (bootstrap) CIs did not span 0. Finally, pairwise contrasts of indirect effects were used to test whether differences in the size of indirect effects were statistically significant (*P*<.05). Analyses were performed with SPSS 23 (IBM Corporation) by using the Process macro [34].

## Results

### Descriptive Statistics and Correlations

Table 1 depicts descriptive statistics for app and smartphone session variables as well as the associations between these variables and gender. Table 2 provides information about the associations among app and smartphone session variables, age, and indicators of tendencies toward SmUD (SAS subscales and total score). Based on the number of weekly app sessions (Table 1), WhatsApp was the most frequently used app in the overall sample, followed by Facebook, Instagram, Snapchat, Messenger, and Telegram.

**Table 1:** The number of weekly sessions on apps and smartphones: descriptive statistics of the overall sample stratified by gender^a^.

App or device			Number of sessions/week, mean (SD)							*U*			*P* value
			All participants (N=124)		Males (n=46)		Females (n=78)						
Facebook			43.25 (65.78)		45.61 (79.78)		41.86 (56.45)		1843.50			.79	
**Image-based social networking apps**			61.81 (104.96)		50.09 (126.24)		68.72 (90.31)		2098.50			.10	
	Snapchat	21.86 (52.03)		22.24 (60.3)		21.64 (46.91)		1841.50			.77		
	Instagram	39.34 (70.35)		27.85 (74.98)		47.08 (66.94)		2098.50			.06		
**Instant messaging apps**			327.31 (219.56)		336.26 (207.92)		322.03 (207.41)		1767.50			.89	
	Telegram	6.41 (40.10)		4.37 (13.88)		7.62 (49.52)		1773.00			.84		
	WhatsApp	319.10 (219.56)		332.96 (226.82)		310.92 (216.24)		1742.50			.79		
	Messenger	8.05 (22.57)		6.57 (15.53)		8.92 (25.9)		1976.00			.31		
Smartphone use			347.26 (199.43)		331.54 (184.20)		356.53 (208.48)		1945.00			.44	

^a^Gender was coded (male=0; female=1).

**Table 2:** The correlations between app and smartphone usage variables and age and between usage variables and the individual differences in tendencies toward smartphone use disorder (N=124).

App or device			Variables, Spearman ρ (*P* values)														
			Age		Life disturbances		Positive anticipation		Withdrawal		CY^a^		Overuse		Tolerance		Total SAS^b^ score
Facebook			−0.06 (.53)		−0.01 (.96)		0.15 (.10)		0.14 (.12)		0.31 (<.001)		0.07 (.48)		−0.01 (.95)		0.14 (.11)
**Image-based social networking apps**			−0.36 (<.001)		0.31 (<.001)		0.29 (.001)		0.27 (.003)		0.40 (<.001)		0.28 (*.*002)		0.35 (.001)		0.40 (<.001)
	Snapchat	−0.33 (<.001)		0.23 (.009)		0.19 (*.*03)		0.23 (.009)		0.30 (<.001)		0.24 (.007)		0.25 (.006)		0.30 (.006)	
	Instagram	−0.26 (*.*003)		0.26 (.003)		0.25 (.006)		0.18 (.049)		0.39 (<.001)		0.22 (.01)		0.30 (<.001)		0.33 (<.001)	
**Instant messaging apps**			−0.26 (.004)		0.18 (.048)		0.33 (<.001)		0.24 (.007)		0.39 (<.001)		0.13 (.15)		0.20 (.03)		0.30 (<.001)
	Telegram	−0.03 (.72)		−0.08 (.37)		0.16 (.07)		−0.03 (.73)		0.13 (.14)		−0.01 (.95)		0.09 (.31)		0.03 (.72)	
	WhatsApp	−0.24 (.006)		0.15 (.10)		0.28 (.002)		0.21 (.02*)*		0.36 (<.001)		0.09 (.33)		0.16 (.08)		0.26 (.003)	
	Messenger	−0.11 (.21)		0.12 (.17)		0.23 (.009)		0.25 (.005)		0.33 (<.001)		0.21 (.02)		0.15 (.10)		0.26 (.004)	
Smartphone use			−0.30 (<.001)		0.23 (.01)		0.38 (<.001)		0.30 (<.001)		0.44 (<.001)		0.20 (.03)		0.23 (.01)		0.38 (<.001)

^a^CY: cyberspace-oriented relationship.

^b^SAS: Smartphone Addiction Scale.

The results of Mann-Whitney U (Table 1) tests indicated that there was no significant association between gender and smartphone and app session variables. With regard to correlations (Table 2), we found that all of the weekly app and smartphone session variables, except those for Facebook (*P*=.53), Messenger (*P*=.21), and Telegram (*P*=.72) sessions, had a significant negative correlation with age. We also analyzed correlations between the objectively recorded SN app, IM app, and smartphone session variables and the indicators of tendencies toward SmUD variables. The daily life disturbances score had positive correlations with the number of Instagram sessions, Snapchat sessions, image-based SN app sessions, IM app sessions, and overall smartphone sessions, but not with the number of Facebook and each specific IM app sessions. Positive anticipation, withdrawal, and the general tendency toward SmUD (ie, the SAS total score) had positive correlations with all of the app and smartphone session variables except for the number of Facebook and Telegram sessions. Cyberspace-oriented relationships had moderate positive correlations with all app and smartphone session variables except for the Telegram session variable. The overuse subscale scores positively correlated with the number of participants’ Snapchat, Instagram, image-based SN app, and Messenger sessions and the number of overall smartphone sessions. Finally, tolerance positively correlated with the number of participants’ Snapchat, Instagram, image-based SN app, and IM app sessions (tolerance did not show significant correlations with sessions on specific IM apps) and the number of overall smartphone sessions.

### Mediation Analyses

We also examined the total effect and mediation models for analyzing the association between the number of smartphone sessions and participants’ general tendency toward SmUD (ie, the SAS total score). The results of total and direct effects are shown in Figure 1. For ease of visualization, the results of control variables (ie, age, gender, and the time of the administration of the SAS) are not shown in the figure but are only discussed in the text. In the total effect model, we found that the number of smartphone sessions had a positive association with participants’ tendency toward SmUD. In the mediation model, after including Facebook, image-based SN app, and IM app session variables in the model, the number of smartphone sessions (ie, screen unlocks) no longer showed a significant association with tendencies toward SmUD. In turn, participants’ number of sessions on image-based SN apps had a significant positive association with tendencies toward SmUD. In contrast, the number of sessions on Facebook and IM apps had no significant association with tendencies toward SmUD. In all tested models, associations between control variables and tendencies toward SmUD were nonsignificant.

The examination of indirect effects (Table 3) showed that the association between the number of smartphone sessions and tendencies toward SmUD was mediated by the number of sessions spent on image-based SN apps (effect=0.010; 95% CI 0.002-0.024; standardized effect=0.090). The remaining indirect effects were not significant. Finally, based on the pairwise contrast of unstandardized indirect effects, there was a significant difference between the indirect effects in the Facebook and image-based SN app session routes (contrast=−0.015; 95% CI −0.032 to −0.001); however, the remaining contrasts were not significant (Facebook vs IM apps contrast: −0.010; 95% CI −0.043 to 0.029; image-based SN vs IM app contrast: −0.005; 95% CI −0.046 to 0.025).

**Table 3:** Indirect effects linking smartphone use to tendencies toward smartphone use disorder stratified by routes of indirect effects.

Route of indirect effects	Effect (95% CI)	Standardized effect (95% CI)
Session on Facebook	−0.005 (−0.016 to 0.006)	−0.041 (−0.132 to 0.049)
Session on image-based apps	0.010 (0.002 to 0.024)	0.090 (0.015 to 0.209)
Session on instant messaging apps	0.005 (−0.029 to 0.036)	0.046 (−0.250 to 0.319)

## Discussion

### Principal Findings

This study investigated the associations between individual differences in self-reported tendencies toward SmUD and a set of objectively measured smartphone activity frequency and IM and SN app usage indicators. First, we explored the hypothesis that objectively measured indicators of smartphone and app usage would have a significant association with self-reported tendencies toward SmUD. Our data support this hypothesis. From a purely quantitative standpoint, based on our findings, the association between actual smartphone usage (assessed via screen unlock events) and overall tendencies toward SmUD is moderate (ρ=0.38) and appears to be close to the upper limit of what has been reported in previous studies that used objective smartphone data [24,25]. Further, our results showed that an indicator of the usage of image-based SN apps (ie, Instagram and Snapchat) had the strongest association with SmUD tendencies when compared with indicators of the use of IM apps and Facebook. It is noteworthy that among the image-based SN apps, the use of Instagram had a relatively stronger association with tendencies toward SmUD variables (ρ: range 0.22-0.39) when compared with the use of Snapchat (ρ: range 0.19-0.30).

The overall use of IM apps was positively related to all of the components of tendencies toward SmUD except for the overuse component. In turn, Facebook use was only associated with the cyberspace-oriented relationship component of SmUD. Moreover, among the different components of tendencies toward SmUD, the cyberspace-oriented relationship score had the strongest association with the usage of both IM and SN apps as well as overall smartphone usage. It should be noted that in our previous work in investigating SmUD among the users of different IM and SN platforms via self-report questionnaires, the highest associations were observed between SmUD and problematic Instagram and WhatsApp use, and the lowest (but still significant) associations were observed between SmUD and Snapchat and Facebook use [20].

We also examined our second hypothesis and tested the potential role of the usage of IM and SN apps as a mediator of the link between smartphone usage (ie, the frequency of screen unlocks) and self-reported tendencies toward SmUD. We go beyond previous studies by exploring the associations between smartphone and app usage variables and self-reported tendencies toward SmUD by using a parallel mediation model. Our findings supported the hypothesis that the association between the frequency of overall smartphone use and tendencies toward SmUD could be mediated by the frequency of the usage of apps that allow for web-based social communication (ie, IM and SN apps). In particular, our analyses indicated the existence of an indirect effect that links smartphone use and tendencies toward SmUD via the image-based SN app session route (ie, the frequency of the use of such apps). However, this indirect effect does not exist in the IM app session and traditional SN app session routes.

Overall, combined with the findings that emerged from the correlation analysis, the results from the mediation analyses are in line with those of previous studies that suggest the importance of social motives for smartphone use as predictors of tendencies toward SmUD [6,8] as well as the relative stronger association between tendencies toward SmUD and the use of image-based SN apps, such as Instagram and Snapchat, when compared with the association between such tendencies and the use of other IM and SN platforms [17]. Further, by highlighting the importance of social motives for both increased smartphone use and SN app use, our findings seem to be in line with a compensation-seeking hypothesis for SN app use (ie, using SN platforms to compensate for perceived social deficits [35]) and, possibly, smartphone use.

### Strengths and Limitations

This study has strengths. Since we used a validated instrument for the assessment of tendencies toward SmUD and smartphone activity data were obtained by the use of an objective assessment procedure (ie, a specifically devised smartphone app), our results appear to be more reliable and valid than those reported by studies that relied solely on self-report measures of smartphone activity. The fact that our results were, in several ways, in line with those reported by previous studies further supports the validity of our findings. Future studies should also use measures for directly assessing individual differences in social networks use disorder and contrast their findings with those that we observed in our study (ie, in the context of tendencies toward SmUD).

This study also has several limitations. First, analyses were performed on a relatively small convenience sample that consisted mostly of young adults aged equal or below 30 years old (116/124, 93.5%). Thus, our results cannot be generalized to the general population. The relatively small sample size may also have had a negative impact on statistical power; based on the posthoc computation, our sample size appears to be large enough to detect medium to large correlations and regression effects, but it was not large enough to detect small associations among the examined variables in a robust way. These limitations are common in psychological studies that are based on objective assessments of smartphone use [23]. Still, replication studies with larger representative samples are needed to improve the quality and generalizability of our results.

### Conclusion

This study provides novel results that shed light on the association between individuals’ use of popular SN and IM apps and tendencies toward SmUD. The results from nonzero correlations and regression analyses seem to suggest that the features implemented in image-based SN apps could be more strongly related to individual differences in tendencies toward SmUD compared to those available in IM apps and traditional SN apps, such as Facebook. Further, we also mention our previous work [20] in using a self-report methodology to demonstrate the robust associations between SmUD and problematic WhatsApp use. However, this study supports the idea that smartphones are just vehicles for accessing many different apps. Our data further indicate that reducing the problematic use of social media and messaging apps (particularly image-based SN app use) might help to reduce the overall SmUD tendencies of a person. Of interest is a recent study [36] that suggests that switching the smartphone mode from color mode to gray mode helps to reduce the time spent on smartphones and social media apps. Image-based SN apps might be particularly less interesting when images are only presented in gray mode.

## Appendix

Multimedia Appendix 1 Spearman correlations among features extracted from smartphone log data, age, and smartphone addiction variables.

## Abbreviations

IM: instant messaging
SAS: Smartphone Addiction Scale
SmUD: smartphone use disorder
SN: social networking

## References

[1] The state of mobile 2020. App Annie.

[2] (2021). Most popular messaging apps. Statista.

[3] Most used social media 2021. Statista.

[4] Marengo D, Poletti I, Settanni M (2020). The interplay between neuroticism, extraversion, and social media addiction in young adult Facebook users: Testing the mediating role of online activity using objective data. Addict Behav.

[5] Montag C, Lachmann B, Herrlich M, Zweig K (2019). Addictive features of social media/messenger platforms and freemium games against the background of psychological and economic theories. Int J Environ Res Public Health.

[6] Elhai JD, Levine JC, Dvorak RD, Hall BJ (2017). Non-social features of smartphone use are most related to depression, anxiety and problematic smartphone use. Comput Human Behav.

[7] Sha P, Sariyska R, Riedl R, Lachmann B, Montag C (2018). Linking internet communication and smartphone use disorder by taking a closer look at the Facebook and WhatsApp applications. Addict Behav Rep.

[8] Zhitomirsky-Geffet M, Blau M (2016). Cross-generational analysis of predictive factors of addictive behavior in smartphone usage. Comput Human Behav.

[9] Montag C, Wegmann E, Sariyska R, Demetrovics Z, Brand M (2019). How to overcome taxonomical problems in the study of internet use disorders and what to do with "smartphone addiction"?. J Behav Addict.

[10] Gao Q, Jia G, Fu E, Olufadi Y, Huang Y (2020). A configurational investigation of smartphone use disorder among adolescents in three educational levels. Addict Behav.

[11] Billieux J, Schimmenti A, Khazaal Y, Maurage P, Heeren A (2015). Are we overpathologizing everyday life? A tenable blueprint for behavioral addiction research. J Behav Addict.

[12] Panova T, Carbonell X (2018). Is smartphone addiction really an addiction?. J Behav Addict.

[13] Leung H, Pakpour AH, Strong C, Lin YC, Tsai MC, Griffiths MD, Lin CY, Chen IH (2020). Measurement invariance across young adults from Hong Kong and Taiwan among three internet-related addiction scales: Bergen Social Media Addiction Scale (BSMAS), Smartphone Application-Based Addiction Scale (SABAS), and Internet Gaming Disorder Scale-Short Form (IGDS-SF9) (Study Part A). Addict Behav.

[14] Brand M, Rumpf HJ, Demetrovics Z, MÜller A, Stark R, King DL, Goudriaan AE, Mann K, Trotzke P, Fineberg NA, Chamberlain SR, Kraus SW, Wegmann E, Billieux J, Potenza MN (2020). Which conditions should be considered as disorders in the International Classification of Diseases (ICD-11) designation of "other specified disorders due to addictive behaviors"?. J Behav Addict. Epub ahead of print.

[15] Carr CT, Hayes RA (2015). Social media: Defining, developing, and divining. Atl J Commun.

[16] Marengo D, Longobardi C, Fabris MA, Settanni M (2018). Highly-visual social media and internalizing symptoms in adolescence: The mediating role of body image concerns. Comput Human Behav.

[17] Noë B, Turner LD, Linden DEJ, Allen SM, Winkens B, Whitaker RM (2019). Identifying indicators of smartphone addiction through user-app interaction. Comput Human Behav.

[18] (2017). #StatusOfMind: social media and young people's mental health and wellbeing. Royal Society for Public Health.

[19] Rozgonjuk D, Sindermann C, Elhai JD, Montag C (2020). Fear of missing out (FoMO) and social media's impact on daily-life and productivity at work: Do WhatsApp, Facebook, Instagram, and Snapchat use disorders mediate that association?. Addict Behav.

[20] Rozgonjuk D, Sindermann C, Elhai JD, Montag C (2021). Comparing smartphone, WhatsApp, Facebook, Instagram, and Snapchat: Which platform elicits the greatest use disorder symptoms?. Cyberpsychol Behav Soc Netw.

[21] Choi J, Rho MJ, Kim Y, Yook IH, Yu H, Kim DJ, Choi IY (2017). Smartphone dependence classification using tensor factorization. PLoS One.

[22] Shin M, Lee K (2017). Measuring smartphone usage time is not sufficient to predict smartphone addiction. Journal of Theoretical and Applied Information Technology.

[23] Ryding FC, Kuss DJ (2020). Passive objective measures in the assessment of problematic smartphone use: A systematic review. Addict Behav Rep.

[24] Rozgonjuk D, Levine JC, Hall BJ, Elhai JD (2018). The association between problematic smartphone use, depression and anxiety symptom severity, and objectively measured smartphone use over one week. Comput Human Behav.

[25] Ellis DA, Davidson BI, Shaw H, Geyer K (2019). Do smartphone usage scales predict behavior?. Int J Hum Comput Stud.

[26] Lee M, Han M, Pak J (2018). Analysis of behavioral characteristics of smartphone addiction using data mining. Appl Sci (Basel).

[27] Montag C, Baumeister H, Kannen C, Sariyska R, Meßner EM, Brand M (2019). Concept, possibilities and pilot-testing of a new smartphone application for the social and life sciences to study human behavior including validation data from personality psychology. J (Basel).

[28] Sariyska R, Rathner EM, Baumeister H, Montag C (2018). Feasibility of linking molecular genetic markers to real-world social network size tracked on smartphones. Front Neurosci.

[29] Messner EM, Sariyska R, Mayer B, Montag C, Kannen C, Schwerdtfeger A, Baumeister H (2019). Insights – Future implications of passive smartphone sensing in the therapeutic context. Verhaltenstherapie.

[30] Insights. insightsapp.

[31] Montag C, Yang H, Elhai JD (2021). On the psychology of TikTok use: A first glimpse from empirical findings. Front Public Health.

[32] Kwon M, Lee JY, Won WY, Park JW, Min JA, Hahn C, Gu X, Choi JH, Kim DJ (2013). Development and validation of a smartphone addiction scale (SAS). PLoS One.

[33] Efron B, Tibshirani RJ (1994). An Introduction to the Bootstrap.

[34] Preacher KJ, Hayes AF (2004). SPSS and SAS procedures for estimating indirect effects in simple mediation models. Behav Res Methods Instrum Comput.

[35] Wegmann E, Brand M (2019). A narrative overview about psychosocial characteristics as risk factors of a problematic social networks use. Curr Addict Rep.

[36] Holte AJ, Ferraro FR (2020). True colors: Grayscale setting reduces screen time in college students. Soc Sci J.

[37] (2018). Gesprächskreis Digitalität und Verantwortung: Austausch zu ethischen Fragestellungen der Digitalisierung. Facebook.

