# Supplementary material for: Exploring the Associations Between Self-reported Tendencies Toward Smartphone Use Disorder and Objective Recordings of Smartphone, Instant Messaging, and Social Networking App Usage: Correlational Study
Source: J Med Internet Res. 2021 Sep 30;23(9):e27093. doi: 10.2196/27093 (PMC8517811; doi:10.2196/27093)
Supplement: Multimedia Appendix 1 [file jmir_v23i9e27093_app1.docx]

Table S1. Spearman Correlations between Features Extracted from Smartphone Log Data, Age and Smartphone Addiction Variables (N = 124)

|  | Variables |  | **1** | **2** | **3** | **4** | **5** | **6** | **7** | **8** | **9** | **10** | **11** | **12** | **13** | **14** | **15** | **16** | **17** |
| --- | --- | --- | --- | --- | --- | --- | --- | --- | --- | --- | --- | --- | --- | --- | --- | --- | --- | --- | --- |
| 1 | Screen On events | **r** | **-** |  |  |  |  |  |  |  |  |  |  |  |  |  |  |  |  |
|  |  | p | - |  |  |  |  |  |  |  |  |  |  |  |  |  |  |  |  |
| 2 | Screen Unlock events | **r** | **0.84** |  |  |  |  |  |  |  |  |  |  |  |  |  |  |  |  |
|  |  | p | <.01 |  |  |  |  |  |  |  |  |  |  |  |  |  |  |  |  |
| 3 | Facebook sessions | **r** | **0.47** | **0.36** |  |  |  |  |  |  |  |  |  |  |  |  |  |  |  |
|  |  | p | <.01 | <.01 |  |  |  |  |  |  |  |  |  |  |  |  |  |  |  |
| 4 | Telegram sessions | **r** | **0.02** | **0.16** | **-0.02** |  |  |  |  |  |  |  |  |  |  |  |  |  |  |
|  |  | p | 0.85 | 0.08 | 0.80 |  |  |  |  |  |  |  |  |  |  |  |  |  |  |
| 5 | WhatsApp sessions | **r** | **0.76** | **0.80** | **0.39** | **0.07** |  |  |  |  |  |  |  |  |  |  |  |  |  |
|  |  | p | <.01 | <.01 | <.01 | 0.48 |  |  |  |  |  |  |  |  |  |  |  |  |  |
| 6 | Messenger sessions | **r** | **0.42** | **0.44** | **0.55** | **0.06** | **0.33** |  |  |  |  |  |  |  |  |  |  |  |  |
|  |  | p | <.01 | <.01 | <.01 | 0.53 | <.01 |  |  |  |  |  |  |  |  |  |  |  |  |
| 7 | Instant Messaging sessions | **r** | **0.79** | **0.84** | **0.42** | **0.14** | **0.98** | **0.40** |  |  |  |  |  |  |  |  |  |  |  |
|  |  | p | <.01 | <.01 | <.01 | 0.13 | <.01 | <.01 |  |  |  |  |  |  |  |  |  |  |  |
| 8 | Snapchat sessions | **r** | **0.40** | **0.36** | **0.31** | **0.11** | **0.38** | **0.19** | **0.40** |  |  |  |  |  |  |  |  |  |  |
|  |  | p | <.01 | <.01 | <.01 | 0.22 | <.01 | 0.03 | <.01 |  |  |  |  |  |  |  |  |  |  |
| 9 | Instagram sessions | **r** | **0.50** | **0.46** | **0.39** | **0.03** | **0.43** | **0.31** | **0.45** | **0.43** |  |  |  |  |  |  |  |  |  |
|  |  | p | <.01 | <.01 | <.01 | 0.73 | <.01 | <.01 | <.01 | <.01 |  |  |  |  |  |  |  |  |  |
| 10 | Image-based SN apps sessions | **r** | **0.56** | **0.54** | **0.40** | **0.03** | **0.51** | **0.31** | **0.53** | **0.72** | **0.89** |  |  |  |  |  |  |  |  |
|  |  | p | <.01 | <.01 | <.01 | 0.77 | <.01 | <.01 | <.01 | <.01 | <.01 |  |  |  |  |  |  |  |  |
| 11 | Age | **r** | **-0.28** | **-0.30** | **-0.06** | **-0.03** | **-0.24** | **-0.11** | **-0.26** | **-0.33** | **-0.26** | **-0.36** |  |  |  |  |  |  |  |
|  |  | p | <.01 | <.01 | 0.53 | 0.72 | 0.01 | 0.21 | <.01 | <.01 | <.01 | <.01 |  |  |  |  |  |  |  |
| 12 | Life disturbances | **r** | **0.20** | **0.23** | **-0.01** | **-0.08** | **0.15** | **0.12** | **0.18** | **0.23** | **0.26** | **0.31** | **-0.25** |  |  |  |  |  |  |
|  |  | p | 0.03 | 0.01 | 0.96 | 0.37 | 0.10 | 0.17 | 0.05 | 0.01 | <.01 | <.01 | <.01 |  |  |  |  |  |  |
| 13 | Positive Anticipation | **r** | **0.31** | **0.38** | **0.15** | **0.16** | **0.28** | **0.23** | **0.33** | **0.19** | **0.25** | **0.29** | **-0.12** | **0.48** |  |  |  |  |  |
|  |  | p | <.01 | <.01 | 0.10 | 0.07 | <.01 | 0.01 | <.01 | 0.03 | 0.01 | <.01 | 0.17 | <.01 |  |  |  |  |  |
| 14 | Withdrawal | **r** | **0.26** | **0.30** | **0.14** | **-0.03** | **0.21** | **0.25** | **0.24** | **0.23** | **0.18** | **0.27** | **-0.29** | **0.61** | **0.60** |  |  |  |  |
|  |  | p | <.01 | <.01 | 0.12 | 0.73 | 0.02 | 0.01 | 0.01 | 0.01 | 0.05 | <.01 | <.01 | <.01 | <.01 |  |  |  |  |
| 15 | Cyberspace-oriented relationship | **r** | **0.37** | **0.44** | **0.31** | **0.13** | **0.36** | **0.33** | **0.39** | **0.30** | **0.39** | **0.40** | **-0.24** | **0.52** | **0.56** | **0.64** |  |  |  |
|  |  | p | <.01 | <.01 | <.01 | 0.14 | <.01 | <.01 | <.01 | <.01 | <.01 | <.01 | 0.01 | <.01 | <.01 | <.01 |  |  |  |
| 16 | Overuse | **r** | **0.19** | **0.20** | **0.07** | **-0.01** | **0.09** | **0.21** | **0.13** | **0.24** | **0.22** | **0.28** | **-0.18** | **0.69** | **0.48** | **0.61** | **0.60** |  |  |
|  |  | p | 0.03 | 0.03 | 0.48 | 0.95 | 0.33 | 0.02 | 0.15 | 0.01 | 0.01 | <.01 | 0.05 | <.01 | <.01 | <.01 | <.01 |  |  |
| 17 | Tolerance | **r** | **0.20** | **0.23** | **-0.01** | **0.09** | **0.16** | **0.15** | **0.20** | **0.25** | **0.30** | **0.35** | **-0.24** | **0.56** | **0.43** | **0.46** | **0.50** | **0.69** |  |
|  |  | p | 0.02 | 0.01 | 0.95 | 0.31 | 0.08 | 0.10 | 0.03 | 0.01 | <.01 | <.01 | 0.01 | <.01 | <.01 | <.01 | <.01 | <.01 |  |
| 18 | Total SAS score | **r** | **0.33** | **0.38** | **0.14** | **0.03** | **0.26** | **0.26** | **0.30** | **0.30** | **0.33** | **0.40** | **-0.28** | **0.77** | **0.75** | **0.84** | **0.79** | **0.82** | **0.72** |
|  |  | p | <.01 | <.01 | 0.11 | 0.72 | <.01 | <.01 | <.01 | <.01 | <.01 | <.01 | <.01 | <.01 | <.01 | <.01 | <.01 | <.01 | <.01 |
